# Febuxostat, a novel xanthine oxidoreductase inhibitor, improves hypertension and endothelial dysfunction in spontaneously hypertensive rats

**DOI:** 10.1007/s00210-016-1239-1

**Published:** 2016-05-20

**Authors:** Takashi Shirakura, Johji Nomura, Chieko Matsui, Tsunefumi Kobayashi, Mizuho Tamura, Hiroaki Masuzaki

**Affiliations:** Pharmaceutical Development Research Laboratories, Teijin Institute for Bio-Medical Research, Teijin Pharma Ltd., 4-3-2, Asahigaoka, Hino, 191-852 Tokyo Japan; Division of Endocrinology, Diabetes and Metabolism, Hematology, Rheumatology (Second Department of Internal Medicine), Graduate School of Medicine, University of the Ryukyus, Nishihara, Okinawa Japan

**Keywords:** Xanthine oxidase, Febuxostat, Endothelial dysfunction, Hypertension

## Abstract

Xanthine oxidase (XO) is an enzyme responsible for the production of uric acid. XO produces considerable amount of oxidative stress throughout the body. To date, however, its pathophysiologic role in hypertension and endothelial dysfunction still remains controversial. To explore the possible involvement of XO-derived oxidative stress in the pathophysiology of vascular dysfunction, by use of a selective XO inhibitor, febuxostat, we investigated the impact of pharmacological inhibition of XO on hypertension and vascular endothelial dysfunction in spontaneously hypertensive rats (SHRs). Sixteen-week-old SHR and normotensive Wistar-Kyoto (WKY) rats were treated with tap water (control) or water containing febuxostat (3 mg/kg/day) for 6 weeks. Systolic blood pressure (SBP) in febuxostat-treated SHR (220 ± 3 mmHg) was significantly (*P* < 0.05) decreased compared with the control SHR (236 ± 4 mmHg) while SBP in febuxostat-treated WKY was constant. Acetylcholine-induced endothelium-dependent relaxation in aortas from febuxostat-treated SHR was significantly (*P* < 0.05) improved compared with the control SHR, whereas relaxation in response to sodium nitroprusside was not changed. Vascular XO activity and tissue nitrotyrosine level, a representative indicator of local oxidative stress, were considerably elevated in the control SHR compared with the control WKY, and this increment was abolished by febuxostat. Our results suggest that exaggerated XO activity and resultant increase in oxidative stress in this experimental model contribute to the hypertension and endothelial dysfunction, thereby supporting a notion that pharmacological inhibition of XO is valuable not only for hyperuricemia but also for treating hypertension and related endothelial dysfunction in human clinics.

## Introduction

Xanthine oxidoreductase (XOR) catalyzes the oxidation of hypoxanthine to xanthine and on to uric acid, which are the final reactions of purine catabolism in humans. Under physiological conditions, the enzyme functions mainly as a dehydrogenase (XDH) and uses NAD^+^ as the electron acceptor. Noticeably, under a variety of pathologic conditions such as tissue ischemia and tissue damage (Harrison 2002), it can be converted into an oxidase (xanthine oxidase (XO)) using molecular oxygen as the electron acceptor.

Because of its ability to generate reactive oxygen species (ROS), the role of XOR has long been investigated in a wide variety of ROS-related diseases (Boueiz et al. 2008).

Hypertension is a major risk for cardiovascular diseases. A large body of evidence suggests that locally exaggerated oxidative stress is profoundly involved in the pathophysiology of hypertension (Nickenig and Harrison 2002; Ceriello 2008; Cutler et al. 2008; Grossman 2008). In accordance with this notion, augmented oxidative stress has been implicated in vascular dysfunction of several models of experimental hypertension ((Fukui et al. 1997; Kerr et al. 1999; Tanito et al. 2004; Touyz 2004). In particular, endothelial dysfunction is a hallmark of hypertension (Lockette et al. 1986; Morawietz et al. 2001; Landmesser et al. 2003), which is also evoked by oxidative stress. As one of underlying mechanisms, it is suggested that oxidative stress reduces bioavailability and bioactivity of nitric oxide (NO), a potent endothelium-derived relaxing factor (Li et al. 2013).

Based on the notion that XO produces ROS, it is reasonable to speculate that XO would ameliorate hypertension and associated endothelial dysfunction. However, conflicting results have been reported depending on the experimental hypertensive models (Tian et al. 2005; Zhang et al. 2005; Yamamoto et al. 2006; Ong et al. 2007; Viel et al. 2008). It also should be noted that most of previous studies used allopurinol to attain the pharmacological XO inhibition. Importantly, allopurinol is a classic suicide inhibitor, as its binding to and reduction of the Mo cofactor induce self-oxidation to form oxypurinol, an active inhibitory metabolite. Reduction of the Mo cofactor by allopurinol leads to ROS production (Galbusera et al. 2006). On the other hand, allopurinol is known to exert radical scavenging effect due to its chemical structure (Augustin et al. 1994). For these reasons, it seems to be hard to precisely evaluate the pharmacological effect of XO inhibition by allopurinol. In contrast, febuxostat is a novel, selective, and potent XO inhibitor with non-purine structure for treating gout in human clinics. Unlike allopurinol, febuxostat does not produce or scavenge ROS by its chemical structure, because it is neither a suicide XO inhibitor nor a radical scavenger (Okamoto et al. 2003). In this sense, febuxostat would be better tool to resolve the issue whether pharmacological XO inhibition reduces blood pressure and improves the endothelial dysfunction in experimental models. To explore the possible involvement of XO-derived oxidative stress in the pathophysiology of vascular dysfunction, we evaluated the impact of febuxostat on hypertension and vascular endothelial dysfunction in spontaneously hypertensive rats (SHRs), representative animal model of human essential or primary hypertension (Zicha and Kunes 1999).

## Materials and methods

### Animals and treatments

Male SHRs and age-matched Wistar-Kyoto (WKY) rats weighing 200–300 g were purchased from Charles River Japan (Yokohama, Japan) and were maintained under standard conditions until the experiments were done. All studies were performed in accordance with procedures approved by the Animal Ethics Committee of the Teijin Institute for Bio-Medical Research Institute, Teijin Pharma Limited, Tokyo, Japan.

Four groups of rats were used in this study: (1) WKY control rats (WKY-C), (2) febuxostat-treated WKY rats (WKY-Fx), (3) control SHR (SHR-C), and (4) febuxostat-treated SHR (SHR-Fx). WKY-C and SHR-C groups were given tap water. The WKY-Fx and SHR-Fx groups were given febuxostat dissolved in drinking water ad libitum at a concentration of 0.03 mg/L. At this concentration, the dose of febuxostat per day calculated by the daily water intake and body weight was approximately 3 mg/kg/day. As a matter of fact, we recently found that single oral administration of febuxostat at 1, 3, and 10 mg/kg to rats showed dose-dependent inhibition of XO activity in plasma and aorta (data not shown). It should be noted that repeated administration of XO inhibitor at high dose in rodents results in death, partly because xanthine calculi caused renal impairment due to inhibition of XOD/XDH (Isa et al. 1968). According to this notion, oral administration of febuxostat at 10 mg/kg for 28 days caused calculi in 1 of 30 animals (Horiuchi et al. 1999). In this context, to achieve the maximal inhibition of XO and avoid possible renal damages, we carefully selected 3 mg/kg/day as a dose of febuxostat. The febuxostat treatment was started at 16 weeks of age and carried out for 6 weeks. Systolic blood pressures (SBPs) were measured before and 2, 4, and 6 weeks during the drug treatment. Vascular function, XO activity, and other biochemical parameters were determined at 6 weeks.

### Blood pressure measurement

SBP was measured using a tail-cuff sphygmomanometer (Visitech BP2000, Visitech Systems, Apex, NC). All animals were acclimated for blood pressure measurements 1 week before drug treatment.

### Xanthine oxidase activity

Plasma and tissue XO activities were measured by the pterin-based assay. In brief, frozen tissues were homogenized with potassium phosphate buffer, pH 7.4, containing 1 mM ethylenediaminetetraacetic acid (EDTA) and protease inhibitors. The homogenates were centrifuged 12,000 rpm for 15 min at 4 °C, and supernatants were co-incubated with 50-μM pterin solution to assay XO activity. After 60-min incubation at 37 °C, fluorometric assays were performed to calculate the production of isoxanthopterin. Activity of purified XO derived from buttermilk (Calbiochem) and protein concentration were measured and used to normalize the sample activity.

### In vitro organ bath studies

The animals were sacrificed at the end of the 6-week study by decapitation under pentobarbital anesthesia. The thoracic aorta was isolated carefully and cut into 3-mm-length ring. The ring segments were mounted in organ baths containing 5 mL of Krebs-Henseleit solution aerated with 95 % O_2_ and 5 % CO_2_ and warmed at 37 °C. The composition of Krebs-Henseleit solution is as follows (mM): NaCl (119), KCl (4.7), CaCl_2_ (2.5), MgSO_4_ (1.2), NaHCO_3_ (25), KH_2_PO_4_ (1.2), and glucose (10); pH7.4. Each ring was connected to a force transducer (FD, NIHON KODEN, Tokyo, Japan) for isometric force recording. The rings were stretched to 2 g of optimal tension and equilibrated for 60 min until a stable baseline tone was obtained.

Following equilibration, the rings were repeatedly exposed to 60 mM KCl, and then, the presence of endothelium was verified by the ability of acetylcholine (ACh, 1 × 10^−6^ M) to relax phenylephrine (PE, 1 × 10^−7^ M)-induced contraction. After washout, endothelium-mediated relaxation was measured as a concentration-response curve to ACh (1 × 10^−10^–1 × 10^−4^ M) in rings contracted with the submaximal dose of PE (5 × 10^−7^ M). Endothelium-independent relaxation was also measured as a concentration-response curve to sodium nitroprusside (SNP) (1 × 10^−10^–1 × 10^−4^ M).

### Oxidative stress measurement

Tissues were weighed and homogenized with 0.05 M potassium phosphate buffer, pH 7.4, containing 1 mM EDTA and protease inhibitors. Homogenates were centrifuged at 12,000 rpm for 15 min at 4 °C, and the supernatants were collected and used for assay. The measurement of nitrotyrosine was performed by using a commercially available nitrotyrosine assay kit (Northwest Life Science, CA, USA) according to the manufacturer’s instructions.

### Plasma uric acid level

Plasma uric acid levels were determined by an enzymatic method based on the uricase-peroxidase system (PUREAUTO^®^ S CRE-N, Sekisui Medical Co. Ltd., Japan).

### Statistical analysis

Data are expressed as mean ± SEM. The responses to ACh and SNP are expressed as percentages of PE contraction. Statistical calculations for significant differences were performed by using Student’s *t* test. Significance was accepted at *P* < 0.05.

## Results

### General conditions of animals

Febuxostat treatment did not alter the body weight in both WKY rats and SHRs. The plasma uric acid levels were similar in WKY-C and SHR-C groups (Table 1). Febuxostat treatment significantly decreased plasma uric acid levels in both WKY rats and SHRs to the same extent (0.9 ± 0.1 mg/dL in WKY-C, 0.5 ± 0.1 mg/dL in WKY-Fx, 1.0 ± 0.1 mg/dL in SHR-C, and 0.5 ± 0.1 mg/dL in SHR-Fx; Table 1).

**Table 1:** Body weight and plasma uric acid

	WKY-C	WKY-Fx	SHR-C	SHR-Fx
BW (g)	376 ± 5	370 ± 6	384 ± 4	387 ± 4
Uric acid (mg/dL)	0.9 ± 0.1	0.5 ± 0.1**	1.0 ± 0.1NS	0.5 ± 0.1##

Values are means ± SEM (*n* = 9∼10 per group)

*NS* not significant WKY-C vs SHR-C, Student’s *t* test

**P < 0.01 WKY-C vs WKY-Fx, ##*P* < 0.01 SHR-C vs SHR-Fx

### Systolic blood pressure

**Fig. 1:**
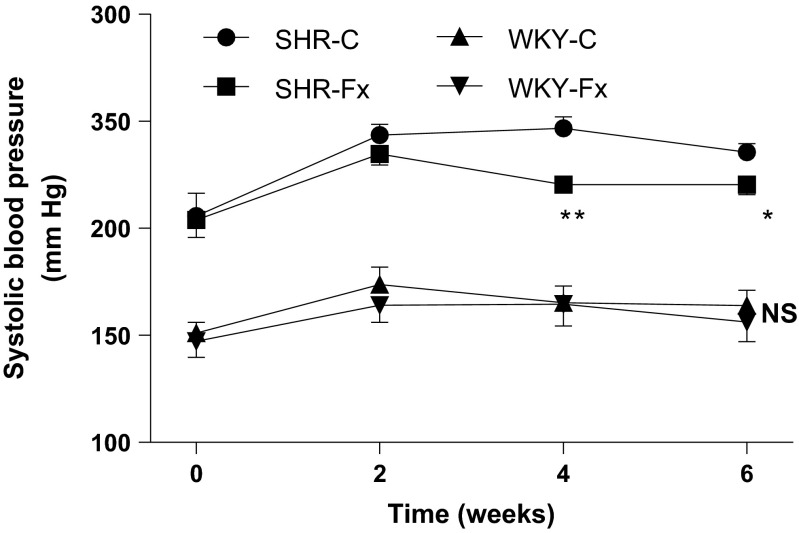
Systolic blood pressure (SBP) in untreated and febuxostat-treated (3 mg/kg/day) spontaneously hypertensive rats and WKY rats. Values are means ± SEM (*n* = 4∼5 per group). *P < 0.05, **P < 0.01 SHR-C vs SHR-Fx, *NS* not significant WKY-C vs WKY-Fx, Student’s *t* test

SBP in the SHR-C group was significantly higher compared to that in the WKY-C group. Febuxostat treatment significantly (*P* < 0.05) decreased SBP in the SHRs (236 ± 4 mmHg in SHR-C, 220 ± 3 mmHg in SHR-Fx, Fig. 1), while it did not alter SBP in the WKY rats after 6-week treatment (164 ± 7 mmHg in WKY-C, 156 ± 9 mmHg in WKY-Fx, Fig. 1).

### Vascular and plasma xanthine oxidase activity

**Fig. 2:**
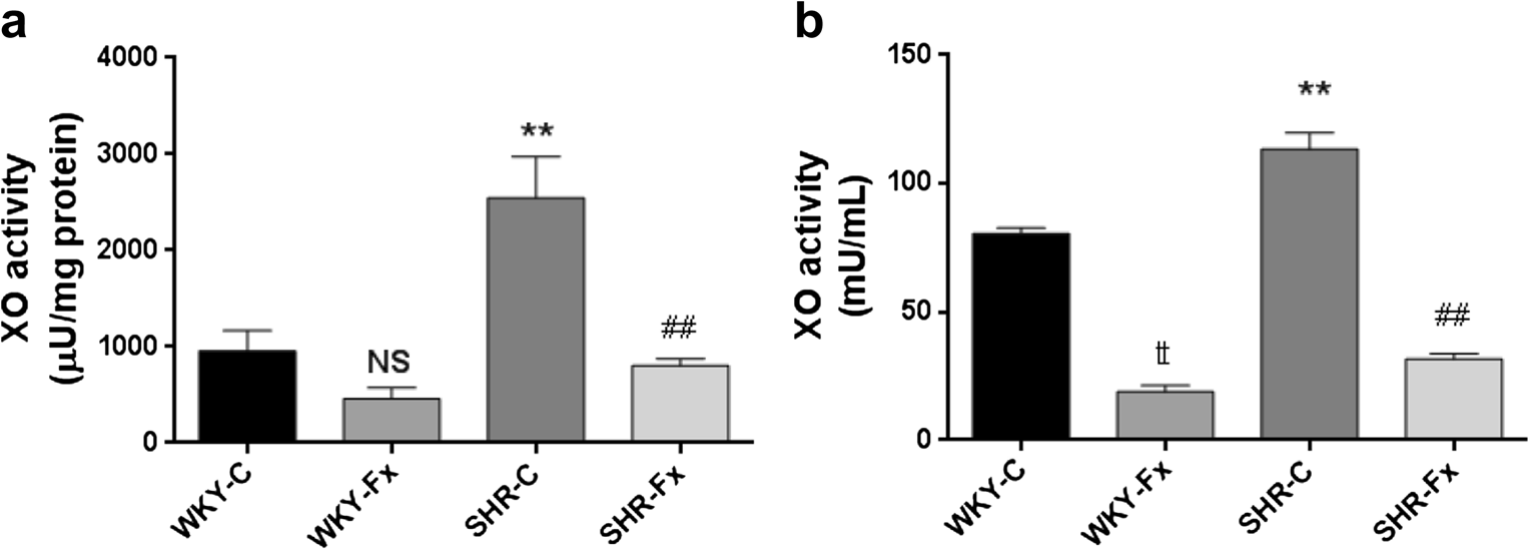
Xanthine oxidase activity in aorta (**a**) and plasma (**b**) obtained from spontaneously hypertensive rats and WKY rats. Values are means ± SEM (*n* = 6 per group). t*P* < 0.05, tt*P* < 0.01 WKY-C vs WKY-Fx, **P < 0.01 WKY-C vs SHR-C, ##*P* < 0.01 SHR-C vs SHR-Fx, Student’s *t* test

XO activity in the thoracic aorta of SHRs was significantly higher than that of WKY rats (Fig. 2a). The treatment with febuxostat lowered this activity in both strains (957 ± 214 μU/mg protein in WKY-C, 463 ± 114 μU/mg protein in WKY-Fx, 2549 ± 427 μU/mg protein in SHR-C, and 805 ± 73 μU/mg protein in SHR-Fx, Fig. 2a). Similarly, the level of XO activity in plasma of SHRs was significantly higher than that of WKY rats (Fig. 2b). Treatment with febuxostat reduced this activity in both strains (80.5 ± 2.2 mU/mL in WKY-C, 19.0 ± 2.5 mU/mL in WKY-Fx, 113.4 ± 6.4 mU/mL in SHR-C, and 31.7 ± 2.1 mU/mL in SHR-Fx, Fig. 2b).

### Vascular oxidative stress

**Fig. 3:**
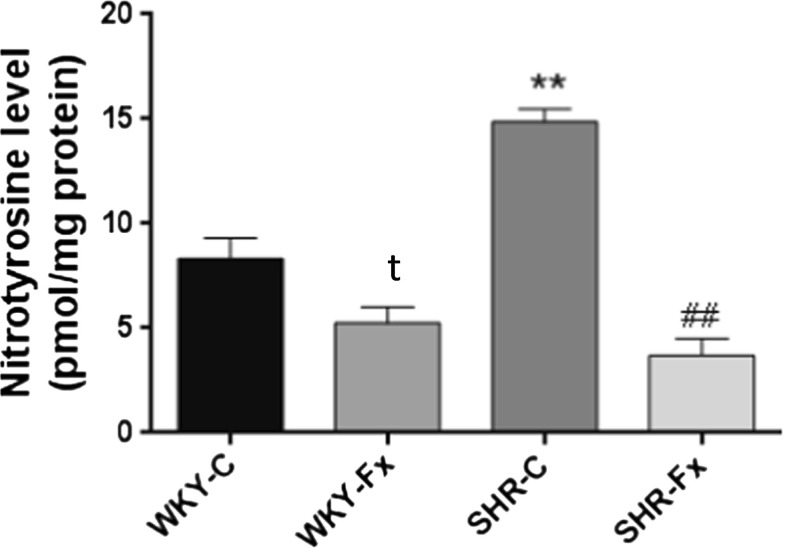
Effect of febuxostat on vascular nitrotyrosine levels in untreated and febuxostat-treated (3 mg/kg/day) spontaneously hypertensive rats and WKY rats. Values are means ± SEM (*n* = 6 per group). t*P* < 0.05 WKY-C vs WKY-Fx, **P < 0.01 WKY-C vs SHR-C, ##*P* < 0.01 SHR-C vs SHR-Fx, Student’s *t* test

To investigate the therapeutic effect of febuxostat on oxidative stress, we examined the tissue nitrotyrosine level. Nitrotyrosine, a marker of nitro-oxidative stress in the thoracic aorta of SHRs, was significantly higher than that of WKY rats (Fig. 3). The treatment with febuxostat lowered nitrotyrosine concentration in both strains (Fig. 3).

### Vascular reactivity

**Fig. 4:**
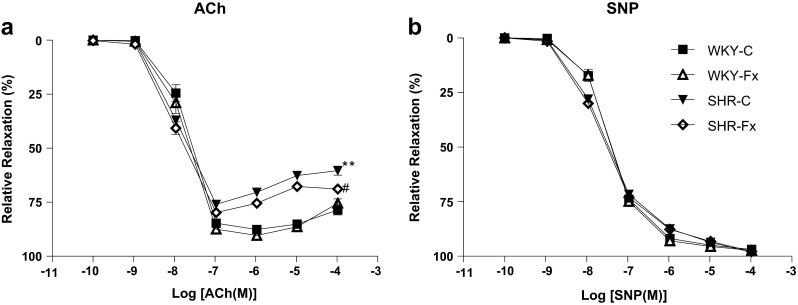
Concentration-response curves to **a** acetylcholine and **b** sodium nitroprusside in aortic rings isolated from untreated and febuxostat-treated spontaneously hypertensive rats and WKY rats. Values are means ± SEM (*n* = 4∼5 per group). **P < 0.01 WKY-C vs SHR-C, #*P* < 0.05 vs SHR-C, Student’s *t* test

Finally, we evaluated the effect of febuxostat on endothelial function. ACh-induced endothelium-dependent relaxation in the thoracic aorta was attenuated in SHR compared to that of WKY rats (*P* < 0.01, Fig. 4a). Febuxostat treatment restored the endothelium-dependent relaxation in comparison to the untreated SHRs (*P* < 0.05, Fig. 4a). The endothelium-independent relaxation measured using SNP, a direct NO donor, was similar in SHRs and WKY rats (Fig. 4b). Febuxostat treatment did not alter the relaxation in response to SNP in both WKY rats and SHRs.

## Discussion

By using a novel, selective xanthine oxidase inhibitor febuxostat, the present study aimed to investigate the therapeutic effects of pharmacological inhibition of XO on hypertension and endothelial dysfunction in SHRs. Our data demonstrated that the therapeutic dose of febuxostat appropriate for hyperuricemia considerably decreased the SBP, reduced the vascular and plasma XO activity, suppressed the vascular nitrotyrosine level, and improved endothelial dysfunction in SHR.

### Improvement in hypertension and endothelial dysfunction

Our results showed that the therapeutic dose of febuxostat appropriate for hyperuricemia significantly ameliorated hypertension in SHR. On the other hand, a couple of studies showed that chronic treatment with allopurinol, a classic type of XO inhibitor, failed to lower blood pressure in SHRs (Trachtman et al. 1991; Laakso et al. 1998, 2004; Yamamoto et al. 2006). With respect to the possible mechanisms for the inconsistent results between the two XO inhibitors, a couple of possibilities are raised as follows. First, allopurinol does produce oxidative stress when metabolized to oxypurinol as described above (Galbusera et al. 2006), while febuxostat does not. Second, both allopurinol and oxypurinol showed the limitation to inhibit the endothelial-binding xanthine oxidase (Kelley et al. 2004; Malik et al. 2011). Third, allopurinol has been shown to be more nephrotoxic in SHR than in WKY, thereby masking its beneficial effect on hypertension (Trachtman et al. 1991). These may explain, at least in part, the difference in impact on hypertension of SHR between allopurinol and febuxostat.

XO inhibitors such as tungsten and allopurinol have been reported to improve endothelial dysfunction in several animal models and human diseases such as atherosclerosis and coronary heart disease (Schroder et al. 2006; Dopp et al. 2011; George et al. 2006; Yiginer et al. 2008). Our recent work demonstrated that febuxostat improved endothelial dysfunction also in high-fat diet-induced obese diabetic mice (Masuzaki et al., manuscript submitted). Based on our results in various experimental hypertension models, XO inhibitors may exert favorable effects in several types of endothelial dysfunction.

### Mechanism of action of XO inhibition

In the present study, XO activities in both aorta and plasma from SHRs were significantly elevated as compared to that of WKY rats. Importantly, treatment of febuxostat substantially lowered aorta and plasma XO activities in both strains. Circulating XO binds to glycosaminoglycan residues on the surface of endothelium in a partially heparin-reversible manner and subsequently translocates to intracellular compartments (Radi et al. 1997; Houston et al. 1999). Although mechanisms whereby plasma XO activity is elevated in SHRs are not yet entirely clarified, it has been reported that XO is released from several organs into systemic circulation in a line of pathophysiology including hepatitis, hemorrhagic shock, sickle cell disease, and ischemia reperfusion (Yokoyama et al. 1990; Tan et al. 1998; Aslan et al. 2001). SHRs represent a variety of organ damages related to severe hypertension (Liu et al. 2011; Boon et al. 2013). Therefore, XO derived from damaged organs may contribute to the elevation of plasma and vascular XO activities in SHRs.

Our data demonstrated that endothelial relaxation by ACh was improved in febuxostat-treated SHRs compared with the vehicle-treated SHRs, while the response to SNP in aorta was unaltered. Enhanced ACh-induced relaxation in vascular endothelium by febuxostat is attributed, at least partly, to the increase in NO response, resulting from the scavenge of NO by ROS, which reacts rapidly with NO to form peroxynitrite (Trujillo et al. 1998). To assess the involvement of NO scavenge in this experimental paradigm, we measured vascular nitrotyrosine levels. Protein nitrosylation is a representative indicator of peroxynitrite formation in vascular tissues. As expected, SHRs showed significantly elevated nitrotyrosine levels in the aortic homogenate as compared to WKY rats, which were normalized by the treatment of febuxostat. Thus, the data suggest that febuxostat attenuates NO scavenge and subsequently increases the availability of NO. Furthermore, peroxynitrite per se induces endothelial and tissue injuries in various pathophysiological conditions (Pacher et al. 2002a, b, 2005; Szabo et al. 2002a, b, 2004; Ungvari et al. 2005). Hence, suppression of peroxynitrite by febuxostat is also beneficial to ameliorate endothelial dysfunction.

It is well documented that elevation of serum uric acid per se is a potent risk for dysfunction of kidney and vascular system, resulting in hypertension (Sundstrom et al. 2005; Galbusera et al. 2006; Krishnan et al. 2007; Grayson et al. 2011). In animal models, hyperuricemia in rats leads to increase in blood pressure, while the alleviation of hyperuricemia with urate-lowering drugs including benzbromarone leads to the reversal of hypertension (Mazzali et al. 2001; Nakagawa et al. 2003; Sanchez-Lozada et al. 2008a, b). In clinical settings, reduction of uric acid with allopurinol in hyperuricemic, hypertensive adolescents did ameliorate blood pressure profile (Feig et al. 2008). In contrast to these findings, the present study used SHRs and the control normotensive WKY rats and compared the results to minimize the possible influence of uric acid-dependent actions, because the serum uric acid level in SHRs is similar to WKY rats (Trachtman et al. 1991; Durante et al. 2010). Treatment of febuxostat equipotently lowers plasma uric acid levels in both WKY rats and SHRs and ameliorated hypertension in SHRs, suggesting that the BP-lowering effects of febuxostat are caused by the suppression of ROS production independent of the lowering effect on plasma uric acid. Comparison study using XO inhibitors and other hypouricemic agents such as benzbromarone would be required to test this hypothesis.

### Study limitations

In the present study, the dose of febuxostat was limited to about 3 mg/kg/day, because the higher dose of febuxostat highly causes xanthine calculi as described (Isa et al. 1968). As a matter of fact, febuxostat at 3 mg/kg/day could not attain the complete inhibition of XO systemically in this study. Therefore, we could not assess the maximal effects of febuxostat on the hypertension and endothelial dysfunction in the current experimental setting. The present study used SHR as a representative animal model of hypertension and demonstrated the potential anti-hypertensive effects by pharmacological XO inhibition. Recently, Boban et al. reported that circulating xanthine oxidase activity was increased in patients with essential hypertension (Boban et al. 2014), which is consistent with our results that plasma XO activity was considerably elevated in SHR. Therefore, XO inhibitor may be beneficial for the treatment of some forms of hypertension in humans. However, there is little information about the relationship between plasma XO activity and other forms of hypertension such as renal hypertension and pulmonary hypertension. In this context, further studies are warranted to clarify the clinical implication of XO inhibition in various types of diseases.

With respect to the prediction of clinical efficacy of XO inhibition, we should be careful about extrapolating the results in experimental animals to humans. Importantly, unlike humans, almost all animals including rodents have uricase, a urate oxidase enzyme. Although few animal species that mimic uric acid metabolism in humans are known (Oda et al. 2002; Bannasch et al. 2008), there seems no available model of hypertension in these species. Therefore, clinical studies are required to verify the effects of XO inhibition on the hypertension and endothelial dysfunction in human pathophysiology.

In conclusion, present study demonstrates that exaggerated XO activity and resultant increase in oxidative stress in SHRs contribute, at least partly, to their hypertension and endothelial dysfunction. This notion was endorsed by the finding that chronic treatment of selective XO inhibitor febuxostat improves a line of vascular phenotypes. Our data suggest that pharmacological inhibition of exaggerated XO may be valuable not only for hyperuricemia but also for treating some forms of hypertension and related endothelial dysfunction in humans.

## Abbreviations

ACh: Acetylcholine
NAD: Nicotinamide adenine dinucleotide
NO: Nitric oxide
ROS: Reactive oxygen species
SBP: Systolic blood pressure
SHR: Spontaneously hypertensive rat
SNP: Sodium nitroprusside
WKY: Wistar-Kyoto
XDH: Xanthine dehydrogenase
XO: Xanthine oxidase
XOR: Xanthine oxidoreductase
